# Physical violence and violent threats reported by Aboriginal and Torres Strait Islander people with a disability: cross sectional evidence from a nationally representative survey

**DOI:** 10.1186/s12889-020-09684-4

**Published:** 2020-11-23

**Authors:** Jeromey B. Temple, Heather Wong, Angeline Ferdinand, Scott Avery, Yin Paradies, Margaret Kelaher

**Affiliations:** 1 Centre for Health Policy, Melbourne School of Population and Global Health, The University of Melbourne, 207 Bouverie St, Carlton, VIC 3053 Australia; 2 First Peoples Disability Network Australia, PO Box A2265, Sydney South, NSW 1235 Australia; 3 School of Social Sciences, Western Sydney University, Sydney, NSW Australia; 4 Centre of Citizenship and Globalisation, Deakin University, 221 Burwood Highway, Burwood, VIC 3125 Australia

**Keywords:** Violence, Threats, Disability, Aboriginal and Torres Strait Islander

## Abstract

**Background:**

A recent Royal Commission into the treatment of Australians living with disabilities has underscored the considerable exposure to violence and harm in this population. Yet, little is known about exposure to violence among Aboriginal and Torres Strait Islander people living with disabilities. The objective of this paper was to examine the prevalence, disability correlates and aspects of violence and threats reported by Aboriginal and Torres Strait Islander people living with disabilities.

**Methods:**

Data from the 2014–15 National Aboriginal and Torres Strait Islander Social Survey were used to measure physical violence, violent threats and disability. Multivariable logistic and ordinal logistic regression models adjusted for complex survey design were used to examine the association between measures of disability and exposure to violence and violent threats.

**Results:**

In 2014–15, 17% of Aboriginal and Torres Strait Islander people aged 15–64 with disability experienced an instance of physical violence compared with 13% of those with no disability. Approximately 22% of those with a profound or severe disability reported experiencing the threat of physical violence. After adjusting for a comprehensive set of confounding factors and accounting for complex survey design, presence of a disability was associated with a 1.5 odds increase in exposure to physical violence (OR = 1.54 *p* < 0.001), violence with harm (OR = 1.55 *p* < 0.001), more frequent experience of violence (OR = 1.55 *p* < 0.001) and a 2.1 odds increase (OR = 2.13 *p* < 0.001) in exposure to violent threats. Severity of disability, higher numbers of disabling conditions as well as specific disability types (e.g., psychological or intellectual) were associated with increased odds of both physical violence and threats beyond this level. Independent of these effects, removal from one’s natural family was strongly associated with experiences of physical violence and violent threats. Aboriginal and Torres Strait Islander women, regardless of disability status, were more likely to report partner or family violence, whereas men were more likely to report violence from other known individuals.

**Conclusion:**

Aboriginal and Torres Strait Islander people with disability are at heightened risk of physical violence and threats compared to Aboriginal and Torres Strait Islander people without disability, with increased exposure for people with multiple, severe or specific disabilities.

## Background

In 2019, the Australian Government established a Royal Commission into the violence, abuse, neglect and exploitation of people living with a disability. The Royal Commission was charged with investigating, among other questions, the prevention, protection and best practise responses to violence and abuse of people living with a disability. One of the key questions raised in the Commission’s issues paper was “What are the experiences of First Nations people with disability regarding violence, abuse, neglect and exploitation”? [1]. In Australia, the Indigenous population consists of the Aboriginal and Torres Strait Islander peoples. Although estimates vary, Aboriginal peoples are thought to have arrived on the Australian continent at least 50,000 years ago, while the Torres Strait Islanders first settled the islands of the Torres Strait approximately 3000 years ago [2, 3]

For many Indigenous peoples worldwide, racism intersecting with colonisation has led to a much higher risk of experiencing violence from both Indigenous and non-Indigenous people across their lifetime [4–6]. In Australia, Aboriginal and Torres Strait Islander people have been subjected to multiple policies of forced removal from land and family, systematic discrimination from education, employment, and services, and currently experience vast inequalities in poverty, health, and overall quality of life outcomes [6–9]. For Indigenous people living with disability, the connection between disability and violence is complex and affected by multiple factors of identity. International studies from the general population show disability and violence are interconnected, as people with disability face increased risk of exposure to violence and experiencing violence can often induce or cause disability [10–13]. This relationship is influenced by the disproportionate poverty and high rates of violence experienced across the life course compared to people without disability, as well as dependence on carers common among people living with disability [14–17]. Exogenously, cultures of silence, encouragement to ignore violence as part of everyday experiences, and imposed norms of helplessness or worthlessness perpetuate cycles of violence [15, 18, 19].

For Indigenous people with disability, racism may intersect with ableism to ultimately create overlapping forms of marginalisation and vulnerability to violence [20]. In Avery’s (2018) study of disability among Aboriginal and Torres Strait Islander people, among 9 of 41 interviews conducted for the research (22%), participants spoke about exposure to violence or a traumatic death of a family member. These included references to close family members who had been murdered or ‘lost,’ gender-based violence, violent attacks, suicide, the death of a young child through medical neglect, and exploitation by a human trafficking ring [20]. This underscores the need for studies that specifically examine the intersection of indigeneity, violence, and disability [21–24]. However, there continues to be a dearth of quantitative research at this intersection. The current literature indicates that the mechanisms that link indigeneity or disability with violence, intersect and amplify for Indigenous people living with disability [25].

In this paper, using nationally representative data, we seek to examine several questions about experiences of physical violence and violent threats reported by Aboriginal and Torres Strait Islander people living with disability. Firstly, are Aboriginal and Torres Strait Islander people living with disability more likely to cite incidents of physical violence or violent threats relative to those without disability? Second, does exposure to violence (physical or threatened) differ by type, severity or numbers of disability conditions? Finally, does the relationship between the perpetrator and victim of violence differ between Aboriginal and Torres Strait Islander people with and without disability?

## Methods

### Data

To answer these research questions, we draw upon data from the National Aboriginal and Torres Strait Islander Social Survey (NATSISS) with in-field operations managed by the Australian Bureau of Statistics (ABS) between September 2014 and June 2015 [26]. The NATSISS includes Aboriginal and Torres Strait Islander people residing in private dwellings and was sampled using a multi-stage design [27]. Following screening, a response rate of approximately 80% was achieved, with this figure marginally higher in remote Australia and marginally lower elsewhere.

Critical to maximizing the survey response rate was the involvement of Aboriginal and Torres Strait Islander people in the design and conduct of the survey. For example, Aboriginal and Torres Strait Islander peak bodies (among others) were engaged at the design stage of the survey. The survey instrument itself varied geographically to ensure questions were culturally appropriate. Of relevance to our present study, the ABS adapted the wording of the concepts of disability and long-term health conditions to account for cultural differences [27]. When a selected respondent was unable to answer questions due to illness or injury, a proxy interviewer was sought when appropriate [27].

Our study is restricted to a sample of *n* = 6417 persons aged between 15 and 64, self-identifying as an Aboriginal or Torres Strait Islander. This age range was used to complement ongoing research on the National Disability Insurance Scheme (NDIS), whose key cliental is within this age group.

NATSISS field operations were conducted under the provisions of the *Census and Statistics Act (CSA) 1905*. Confidentialised data and access to the Remote Access Data Laboratory (RADL) were made available to the authors for this study through the ABS and Universities Australia agreement. Ethics approval for our analyses was granted by the Melbourne School of Population and Global Health Human Ethics Advisory Group (HEAG) – Ethics ID: 1953628.1.

### Measures

#### Disability

The ABS defines disability as “any limitation, restriction or impairment which restricts everyday activities and has lasted, or is likely to last, for at least six months” [27]. The 2014–15 NATSISS operationalises this definition utilizing the short disability module (SDM). The SDM includes a number of measures to identify a health condition that is ongoing and restricts day-to-day activities. For example, in Module 10.02 of the NATSISS, respondents are queried with a series of prompt cards as to the presence of a series of conditions. For those responding yes, they are further prompted “Are you restricted in everyday activities because of this health condition”. Measurement of co-morbidities is included, and respondents are further requested to indicate specific conditions that “are likely to last or have lasted, for six months or more”. The ABS notes that “the full SDM was used in both non-remote and remote areas, with some wording amendments to aid comprehension” [27]. ABS datasets utilizing the SDM show a higher prevalence of disability relative to the more detailed Survey of Disability and Carers. See ABS (2019) and Temple et al. (2020) for a discussion on disability measurement across ABS surveys [28, 29].

Following other studies, we utilize several measures of disability [29]. First, a single measure (Yes/No) indicating the presence of any disability. Second, a measure of the severity of disability, defined by the ABS (as profound or severe, moderate or mild, other restrictions as defined by the level of assistance needed with tasks such as self-care). Third, a measure of the type of disability including (1.) sight, hearing or speech, (2.) physical, (3.) intellectual, (4.) psychological, (5.) head injury, brain damage or stroke, (6.) other restrictions. Finally, a measure of the number of disability types was included.

#### Violence and violent threats

In the NATSISS, physical violence refers to “any incident that involves physical assault, which is the use of physical force by a person with the intent to harm or frighten another person. It includes being pushed, shoved, hit or attacked with a weapon. Other forms of abuse (e.g. sexual, emotional, psychological) are not included” [27]. The question used to measure physical violence was: “In the last 12 months, did anyone, including people you know, use physical force or violence against you?”. Respondents reporting yes were asked the frequency of violence (in the last 12 months) and how they knew the perpetrator. Interviewers used a prompt card (Prompt card S1) to elicit the relationship as follows: current partner, previous partner, ex-boyfriend or ex-girlfriend, parent, child, sibling, other family member, friend, work colleague/fellow school student, neighbor, known by sight only or other known person. For physical threats, respondents were asked “In the last 12 months, did anyone, including people you know, try to use or threaten to use physical force or violence against you?”. There is the possibility that due to the presence of the perpetrator (in the case of family violence), that the respondent may not feel comfortable in disclosing instances of violence. As part of the field operations, the ABS notes: “In order to conduct a personal interview with the selected person (i.e., the respondent), interviewers made appointments to call-back to the household, as necessary. All interviews were conducted face-to-face. Due to the sensitive nature of the survey questions, it was suggested that interviews be conducted in private. However, interviews may have been conducted in private or in the presence of other household members, according to the wishes of the respondent” [27].

### Statistical tests

All statistical tests for this study were conducted using Stata via the ABS Remote Access Data Laboratory [26]. Firstly, simple weighted percentages of violence were disaggregated by the measures of disability outlined above. Following, a series of multivariable logistic and ordinal logistic regression models were fitted to measure the association between disability measures and exposure to violence. Following previous studies, separate regression models were estimated for each disability type. E.g., for the physical disability model, a categorical variable measuring no disability, physical disability, disability other than physical were included [29]. The NATSISS microdata file included 250 replicate weights to account for non-response and sample design features.

As violence is a relatively rare event, previous studies have adopted relatively parsimonious models with few controls included. For example, in two recent Australian studies of violence and disability in the non-Indigenous population, controls are included for age and sex [30, 31]. In a UK study, adjustments are made for age and gender as well as socio-economic disadvantage and neighbourhood quality [32]. Dammeyer and Chapman’s 2018 study of disability violence in Denmark adjusts their analysis for gender, age and presence of either a physical or mental disability [33]. For our study of Aboriginal and Torres Strait Islander people, it is also important to include additional controls. Berry et al. (2009) find hospitalisation rates because of interpersonal violence increase with geographic remoteness [6]. Other studies note the importance of removal from natural family as an important factor heightening exposure to violence for Indigenous peoples worldwide [7, 34–36]. A recent Australian study also highlights a strong association between removal from natural family and racism [29] and another notes the increased prevalence of family removal for Aboriginal and Torres Strait Islander people living with a disability [37].

Following these studies, we present both adjusted and unadjusted odds ratios to examine the association between measures of disability and exposure to violence. The unadjusted odds ratios (OR) are calculated using only age and sex (in addition to the relevant disability measure) in each model. The fully adjusted odds ratios (AOR) included a full list of control variables including:

- Age (15–29, 30–44, 45–54, 55–64)
- Sex (male, female)
- Remoteness (resides in remote Australia, does not reside in remote Australia). Remote Australia is defined by the Australian Statistical Geography Standard [27].
- Household Income (0–19%, 20–39%, 40–59%, 60–79%, 80–100%, missing). Specifically, this is gross household income, adjusted using an equivalence scale and coded into the income distribution bands defined above.
- Marital Status (not married, married)
- Removal from natural family (no, yes). This was measured by asking “Have you been removed from your family by welfare or the government or taken away to a mission?”.

The need to estimate parsimonious models is pronounced due to 1) the rarity of violent events, and 2) the need to employ a jack-knife procedure on 250 separate replicate samples to calculate correct variance estimates for the regression coefficients and odds ratios. A further consequence of these design constraints is that there is insufficient sample size to split the regression analyses by other characteristics such as gender, due to matrix conformability issues. Nonetheless, we present selected split sample characteristics by gender in the descriptive statistics. Moreover, following the multivariable results, we present weighted descriptive statistics stratified by measures of disability and other characteristics on the relationship of the victim of physical violence to the perpetrator in the last act of violence.

## Results

Before turning to the key research questions, it is useful to point to the characteristics of this sample of Aboriginal and Torres Strait Islander people aged 15–64 (Table 1). Reflective of the younger age structure of the Indigenous population (relative to the non-Indigenous population), approximately 45% of respondents were aged under 30 and about 10% aged between 55 and 64. The vast majority of respondents (78%) resided in non-remote Australia and the gender split is relatively equal. Just over 40% of respondents were married (41.6%) or living with a disability (43.3%).

**Table 1:** Models of Violence and Disability, Binary Disability Measure

		Physical Violence			Frequency Physical Violence				Violence with Harm			Violent Threats			Sample Characteristics
		W(%)	OR	OR(A)	W(%)	W(%)	OR	OR(A)	W(%)	OR	OR(A)	W(%)	OR	OR(A)	W(%)
					1–2 times	3+ times									
*Disability*															
No		11.7	–	–	7.7	4	–	–	6.3			13.8			56.7
Yes		16.9	1.73***	1.54***	10	7	1.75***	1.55***	9.8	1.77***	1.55**	24.4	2.28***	2.13***	43.3
Control Variables															
*Age*															
15–29		16.1	–	–	9.8	6.3	–	–	8.6	–	–	20.2	–	–	45.6
30–44		14.7	0.84	0.96	8.4	6.3	0.84	0.95	9	0.98	1.18	19.3	0.85	0.94	28.6
45–54		11.2	0.58***	0.59**	8.4	3.1	0.57***	0.59**	6.2	0.62*	0.63*	16.5	0.64**	0.69*	15.7
55–64		6.7	0.32***	0.32***	5.4	1.3	0.31***	0.32***	3.3	0.30**	0.31**	10.3	0.35**	0.38***	10.1
*Sex*															
Male		13.4	–		8.9	4.5	–	–	7	–	–	18.9	–	–	48.1
Female		14.5	1.08	1.02	8.5	6.1	1.09	1.04	8.5	1.21	1.15	17.8	0.89	0.83	51.9
Married															
No		17.1		–	10.1	7		–	10		–	21.1		–	58.4
Yes		9.6		0.60***	6.7	2.9		0.59***	4.6		0.50***	14.5		0.69**	41.6
Income															
0–19%		8.2		–	6.9	1.3		–	4.6		–	13.6		–	8.2
20–39%		8.8		0.98	5.7	3.1		1	4.4		0.88	16.1		1.1	13.8
40–59%		12.1		1.43	7.7	4.4		1.44	7.2		1.49	17		1.21	19.3
60–79%		17.6		1.90*	9.9	4.4		1.95*	10.1		1.82	22.2		1.48	19.8
80–100%		19.2		2.0*	11.8	7.4		2.01*	12.1		2.06+	21		1.3	13.6
Missing		14.5		1.64	9.1	5.4		1.66+	7		1.28	17.7		1.17	25.4
Remote Australia															
No		13.6		–	8.1	5.5		–	8		–	18.9		–	78.3
Yes		15.3		1.23+	10.8	4.5		1.2	7.1		0.94	16.3		0.86	21.7
Family Removal															
No		13		–	8.3	4.7		–	7.2		–	17.4		–	90.6
Yes		23.4		1.98***	11.8	11.6		2.0***	12.7		1.75**	27.7		1.70***	9.4
*Full Population*		14	n.a.	n.a.	8.7	5.3	n.a.	n.a.	7.8	n.a.	n.a.	18.3	n.a.	n.a.	n.a.

Notes: W(%) - weighted percentage; OR - odds ratio adjusted by age and sex; OR(A) odds ratio adjusted by all control vairables; ***p < 0.001 **p < 0.01 *p < 0.05 + *p* < 0.10. n.a. not applicable

### Violence by disability and selected characteristics

Table 1 disaggregates the weighted prevalence (W%), odds ratios adjusted by age and sex (OR) and odds ratios adjusted by all covariates (ORA) for physical violence, frequency of physical violence, violence with harm and violent threats. Across all measures of violence, people with a disability were at a heightened risk compared to people without a disability. Overall, about 17% of those with a disability reported experiencing an act of physical violence in the last 12 months, compared with just under 12% of those without a disability. In line with these results, people living with a disability were also more likely to report higher frequency of physical violence as well as violence with harm. Just under one quarter of people with a disability reported being subject to violent threats in the past year, compared with 14% of those without a disability.

These descriptive statistics point to an association between disability and exposure to violence. However, it is important to control for confounding factors and to adjust for the complex sampling design of the NATSISS. As can be seen in Table 1, a number of factors independent of disability appear to be associated with violence. For example, prevalence tends to decrease with age and marriage, but increase with decreasing income, family removal and marginally, residence in a remote part of Australia. Controlling for these factors we find that disability is associated with a 1.55 to 1.75 fold increase (dependent on controls included) in the odds of reporting physical violence (ORA 1.54 *p* < 0.001), more frequent physical violence (ORA 1.55 *p* < 0.001) and violence with harm (ORA 1.55 *p* < 0.001). Disability was associated with a doubling of odds of violent threats (ORA 2.13 *p* < 0.001). Although not the key purpose of this study, it is noteworthy that family removal was associated with an approximately doubling of odds for experiencing physical violence, more frequent physical violence, violence with harm, and threats of violence (ORA 1.98 *p* < 0.001, ORA 2.0 *p* < 0.001, ORA 1.75 *p* < 0.01, ORA 1.70 *p* < 0.001 respectively).

Table 2 re-estimated the above models by measures of the severity of disability, type of disability and number of disability types reported. Importantly, these results show that severity of disability is associated with exposure to violence. Aboriginal and Torres Strait Islander people with profound or severe disability were in excess of double the odds or risk of physical violence (ORA 2.24 *p* < 0.001), more frequent violence (ORA 2.17 *p* < 0.001), violence with harm (ORA 2.30 *p* < 0.001) and violent threats (ORA 2.52 *p* < 0.001), relative to those with no disability. The odds ratios for those with other restrictions were lower. This is reflected in the raw prevalence rates, showing just under one quarter of people with severe or moderate disability reported physical violence (and just under 30% violent threats) compared with 16.7% of those with moderate or mild disability and 15.5% of those with other restrictions related to self care, mobility, and/or communication tasks.

**Table 2:** Models of Violence and Disability, Detailed Disability Measures

	Physical Violence			Frequency Physical Violence				Violence with Harm			Violent Threats		
	W(%)	OR(P)	OR(A)	W(%)	W(%)	OR(P)	OR(A)	W(%)	OR(P)	OR(A)	W(%)	OR(P)	OR(A)
				1–2 times	3+ times								
No Disability	11.7	–	–	7.7	4	–	–	6.3	–	–	13.8	–	–
**Severity of Disability**													
Profound/Severe	22.1	2.50***	2.24***	15.7	6.4	2.43***	2.17***	13.4	2.66***	2.30**	27.6	2.81***	2.52***
Moderate/Mild	16.7	1.92***	1.59**	9	7.7	2.01***	1.64**	10.5	2.20***	1.76**	24.5	2.59***	2.31***
Other Restrictions	15.5	1.45**	1.35*	8.8	6.7	1.47**	1.36*	8.2	1.37+	1.28	23.2	2.01***	1.96***
**Type of Disability**													
Sight/hearing/speech													
b.) yes	16.5	1.77***	1.57**	9.8	6.7	1.80***	1.59**	9.3	1.80**	1.60*	24.7	2.43***	2.32***
c.) no, but with disability	17.3	1.70***	1.52**	10.2	7.1	1.71***	1.52**	10.1	1.75***	1.52*	24.1	2.16***	2.00***
Physical													
b.) yes	18.1	1.98***	1.72***	10.3	7.8	2.02***	1.75***	10.7	2.07***	1.76***	24.2	2.36***	2.18***
c.) no, but with disability	15	1.39*	1.30+	9.5	5.6	1.40*	1.3	8.1	1.38+	1.28	24.6	2.15***	2.07***
Intellectual													
b.) yes	24.2	2.47***	2.17***	14.1	10	2.55***	2.22***	14.7	2.62***	1.73**	28.5	2.57***	2.36***
c.) no, but with disability	15.3	1.54***	1.39**	9	6.2	1.55***	1.39**	8.6	1.57**	2.09**	23.4	2.20***	2.07***
Psychological													
b.) yes	24.5	2.69***	2.34***	13.7	10.9	2.76***	2.42***	17	3.23***	2.71***	35.8	3.92***	3.64***
c.) no, but with disability	15	1.50**	1.35*	9.1	5.9	1.52**	1.35*	7.9	1.42*	1.26	21.4	1.93***	1.81***
Head injury/stroke/brain damage													
b.) yes	26.8	3.74***	2.67**	16.1	10.7	3.95***	2.76**	23.5	6.14***	4.17***		2.30*	1.83
c.) no, but with disability	16.7	1.69***	1.52***	9.8	6.8	1.71***	1.53***	9.4	1.70***	1.50**		2.28***	2.14***
Other restrictions													
b.) yes	19.9	2.37***	2.01***	10.7	9.2	2.43***	2.07***	12.7	2.64***	2.13***	28.8	3.24***	2.88***
c.) no, but with disability	15.8	1.54***	1.41**	9.7	6.1	1.55***	1.41**	8.6	1.52**	1.38*	22.6	2.01***	1.93***
**Count of Disabilities**													
Count		1.38***				1.40***			1.44***			1.46***	1.38***

Notes: W(%) - weighted percentage; OR - odds ratio adjusted by age and sex; OR(A) odds ratio adjusted by all covariates in Table 1; ***p < 0.001 **p < 0.01 *p < 0.05 + *p* < 0.10

As a further proximate measure of the severity of disability, each additional disability type increased the odds of violence and violent threats between 1.4 and 1.5 times. Results in Table 2 also provide insights into the types of disabilities associated with violence and violent threats. Although the levels of exposure to violence were elevated for people with all disability types, the risk was particularly acute for people with intellectual disabilities; psychological disabilities; and those with head injury, stroke or brain damage related disabilities. People with psychological disabilities and those with head injury, stroke or brain damage related disabilities showed a 2.5 to 4-fold increase in the odds of reporting any form of violence or violent threats.

### Victim-perpetrator relationship

The NATSISS includes additional detail about contextual aspects of physical violent events and selected detail on physical threats. Table 3 displays the relationship of the victim to perpetrator, by selected disability measures and gender. Tests of proportions between gender samples are also included in the table.

**Table 3:** Victim Perpetrator relationship, by Gender and Disability Status, Weighted %

	Males				Females							Total				
	Relationship to Perpetrator				Relationship to Perpetrator							Relationship to Perpetrator				
	Intimate Partner	Family	Other Known	Unknown	Intimate Partner		Family		Other Known		Unknown		Intimate Partner	Family	Other Known	Unknown
**Disability**																
No	10.3	7.6	62.4	19.7	41.3	**	17.1		35.1	***	6.6		26.9	12.7	47.8	12.6
Yes	4.6	13.3	59.9	22.1	40.0	***	17.5		37.3	***	5.2	+	24.3	15.7	47.4	12.7
**Severity of Disability**																
Profound/Severe	0.0	7.2	68.4	24.5	30.1	**	19.2		49.4	***	1.3	*	19.6	15.0	56.0	9.4
Moderate/Mild	5.4	3.4	66.6	24.6	38.1	**	23.4	***	33.2	***	5.3	*	24.4	15.0	47.2	13.4
Other Restrictions	5.7	21.3	53.2	19.8	47.9	***	11.5		33.1	***	7.5		26.5	16.5	43.3	13.8
**Type of Disability**																
Sight/hearing/speech																
b.) yes	5.3	10.6	64.0	20.1	26.1	*	16.7		54.0	+	3.2	*	16.0	13.7	58.9	11.4
c.) no, but with disability	4.0	16.0	56.0	24.0	50.0	***	18.1		25.3	***	6.6	+	31.1	17.3	37.9	13.8
Physical																
b.) yes	6.1	11.8	56.9	25.2	37.9	**	19.6		37.0	***	5.5	*	22.9	15.9	46.4	14.8
c.) no, but with disability	< 1	17.1	67.7	14.3	43.7	***	14.0		37.8	***	4.6		27.2	15.2	49.3	8.3
Intellectual																
b.) yes	4.3	7.3	73.9	14.5	40.5	***	13.9		41.8	***	3.8		25.0	11.1	55.6	8.4
c.) no, but with disability	4.8	15.9	54.1	25.3	39.7	**	19.2		35.3	***	5.8	*	24.0	17.7	43.7	14.6
Psychological																
b.) yes	10.6	2.5	64.8	22.1	47.2	**	14.0	+	31.2	***	7.6		32.1	9.3	45.1	13.6
c.) no, but with disability	1.9	18.3	57.7	22.1	35.9	**	19.5		40.8	**	3.8	*	20.3	19.0	48.6	12.2
Head injury/stroke/brain damage																
b.) yes	12.8	10.4	67.9	8.9	25.0		0.0	**	57.3	**	17.8		16.9	6.9	64.3	11.9
c.) no, but with disability	4.0	13.6	59.3	23.2	40.5	***	18.1		36.7	***	4.8	*	24.7	16.1	46.5	12.7
Other restrictions																
b.) yes	6.1	13.7	53.9	26.4	31.1	*	24.9		39.7	**	4.3	*	20.7	20.3	45.6	13.5
c.) no, but with disability	3.9	13.1	63.0	19.9	45.4	***	13.0		35.9	***	5.7		26.3	13.1	48.4	12.3

Notes: Weighted percentage; − Male is the comparison category for test of proportions; ***p < 0.001 **p < 0.01 *p < 0.05 + *p* < 0.10

An important finding of this research is the differences in the relationship of the victim to the perpetrator by gender. Whereas overall physical violence did not differ by gender in these data, women regardless of disability status were significantly more likely to report partner violence relative to men. ‘Partner violence’ here refers to violence perpetrated by a current or previous partner; boyfriend, girlfriend or date; or ex-boyfriend or ex-girlfriend. Similarly, levels of other familial violence (family relationships other than partners) was also higher for women. Men, on the other hand again, regardless of disability status were more likely to report other known persons (e.g., friends etc), or unknown persons as the perpetrator of violence. Within gender groups, males with a disability were slightly less likely to cite partner violence and more likely to report other familial violence than women with a disability. Nonetheless, the differences in the relationship with the perpetrator are more pronounced between gender groups, rather than on the basis of disability presence alone.

## Discussion

Motivated by the ongoing Royal Commission into violence, abuse, neglect and exploitation of people living with a disability, we sought to examine the exposure of Aboriginal people living with disabilities to violence. Our study found that within the Aboriginal and Torres Strait Islander population, presence of a disability was associated with a 1.5 fold increase in the odds of exposure to physical violence, a higher frequency of violence, and doubling of the odds of experiencing threats of violence. These findings are consistent with quantitative evidence showing people with disability and Indigenous people are at much higher risk of experiencing violence than those without disability or non-Indigenous people [5, 6, 11, 16, 38–40].

Qualitative studies have theorised that when the two identities intersect, the systems of disadvantage that Indigenous people and people with disability navigate overlap to greater effect [25]. In Australia, narrative research on the experiences of Aboriginal and Torres Strait Islander people with disability found that more than a fifth of interviews referenced exposure to violence or a traumatic death of a close family member [20]. This is despite participants not being asked directly about exposure to violence. The high prevalence of Indigenous family and racially-motivated violence, incarceration, and poverty, in conjunction with the overrepresentation of people with disability in carceral settings and institutions, all work to increase risk of exposure to violence [21].

This is in addition to other external factors which increase likelihood of remaining in violent situations: disability often being underdiagnosed due to race, Indigenous spaces not having sufficient funding for accessibility measures, and the siloing of organisations into distinct Indigenous and disability-specific services [8]. The overall high prevalence of violence against people with disability is thought to be the result of lack of economic opportunity which increases the likelihood of living in areas with elevated crime rates and leads to the increased dependence on carers that people with disability experience, in combination with the colonial racism that has led to systemic poverty and trauma [41]. Cripps and Adams (2014) further conceptualise the factors contributing to family violence in Aboriginal communities into two key factors [42]. The first are factors attributable to colonisation, including dispossession and cultural dislocation, family removal and policies and practices that continue to impact Aboriginal people, increasing the risk of violence. The second group of factors increasing the risk of family violence in Aboriginal communities relate to those that can occur in any vulnerable population. For example, unemployment and welfare dependency, destructive coping behaviours, mental health issues and past instances of violence or abuse. Evidence also suggests that these latter factors indicative of poor socio-economic outcomes are also experienced by people living with disabilities in the broader Australian population – for example, lower levels of education, employment, housing vulnerability and deleterious financial wellbeing [43, 44].

The findings from this study that show an elevated risk of experiencing violence based on severity and type of disability are in line with the wider literature regarding levels of violence experienced by people with disability. Severity of disability is strongly associated with all measures of violence. Aboriginal and Torres Strait Islander people with profound or severe disability were at a minimum 2-fold increase in odds of reporting each measure of violence and almost 3 times more likely to be exposed to violent threats compared to Aboriginal and Torres Strait Islander people without a disability. As a further proxy measure of the severity of disability, multiple disability types increased the odds of exposure to violence by approximately 1.4–1.5 times with each successive disability type.

This association is thought to be the result of the contribution of severity of disability to inability to leave violent situations and how that vulnerability contributes to the power dynamics of violence. Those with more severe disabilities are inherently more reliant on carers, thus are more likely to stay in violent carer relationships and be physically unable to escape violent situations [45, 46]. They also face greater barriers to communicating to authority figures that they have experienced violence, among them being limited verbal communication or not being believed as a result of their disability [39]. These vulnerabilities make it more likely that a person with violent or controlling intentions seek out people with disability [47].

Violence was also more pronounced for those living with intellectual disability, psychological disability and/or head injury/stroke/brain damage compared to people with other types of disabilities. In a national-level Danish study, it was also found that people with learning or mental disability were significantly more likely than people with physical disability to experience violence, harassment, or abuse [33]. A systematic review and meta-analysis of risk of violence against adults with disability found an increased risk for people with intellectual impairments compared to those with non-specific impairments; those with mental illnesses were found to have a higher risk than both other categories [11]. Similarly, in the UK, the risk of exposure to violent crime was elevated among adult with mental health problems in comparison to both adults with no disability as well as those with other forms of disability [16]. Interpreting this finding, it is also important to note that disabilities such as those related to head injury may in themselves be caused by a violent event [48]. Evidence from hospital morbidity data suggests that Aboriginal people, in particular Aboriginal women, are at a significantly increased risk of head injury due to assault [49].

Family removal, the practise of intentionally taking Indigenous children from their natural family and placing them in other settings, was found in the analysis to be a significant predictor of experiencing violence [34]. This practise has historically been documented in Australia, Canada, and the USA under the names of the “Stolen Generations”, “Residential Schools”, “the Sixties Scoop”, and “residential boarding schools”, in which the goal was to deny access to Indigenous heritage in order to assimilate Indigenous children into dominant cultures [34, 36, 50]. It has been theorised both as inherently violent by breaking family ties and connections with community, and as a pathway to experiencing violence in care institutions or foster homes [51, 52]. Removed children, regardless of disability status, experienced high rates of physical, sexual, and emotional violence in their new care settings [36]. This was in addition to the constant degradation of their Indigenous identity and attempts to break ties to their culture [53]. Survivors also report poor housing, food, and educational quality within their care institutions [51, 52]. Family removal currently manifests in foster or government care processes. It can include efforts to maintain connections to culture and heritage, such as placing children with other Indigenous families, but remains a traumatic experience for children and their families and is experienced at significantly elevated rates by Aboriginal and Torres Strait Islander people [54].

The impacts of experiencing family removal are wide-reaching and drastic. Survivors of family removal have an increased likelihood of being arrested or being charged with an offence, having alcohol or illicit drug use disorders, cancer, diabetes, heart disease, stroke, back and eye conditions, being unemployed or dependent on government payments, attempted suicide or having suicidal thoughts, worse educational outcomes, cognitive, behavioural or emotional disability, being a victim of violence, loss of parenting skills, living with an infectious disease, or having a mental illness [7–9, 35, 50, 55–57]. All of these factors increase barriers to socioeconomic mobility, which in turn, increase the likelihood of living in an area with higher crime [32]. Lower socio-economic mobility additionally limits the ability to escape violent situations by decreasing financial stability [32].

We further found that Aboriginal and Torres Strait Islander women, regardless of disability status, were more likely to report family violence, whereas men were more likely to report violence from other known individuals (i.e., non-partner or familial). The gendered sources of violence have been found in a national analysis of American crime victimisation, in which men were more likely to experience violence from a relative, whereas women were more likely to experience violence from an intimate partner [17]. The same was true in the Australian setting in a non-Indigenous-specific study [31]. Women with disability are considered to have a higher risk of experiencing intimate partner or family violence, as compared to men with disability. This is in line with other Australian research on the experiences of Aboriginal and Torres Strait Islander people with disability, where 7 of 9 interviews referencing experiences with violence were with women [20]. A potential explanatory factor is the socialisation of women with disability to believe that they are uniquely dependent on their partner for long-term care and economic stability, to be compliant, and that they are unable to access caring, loving relationships [39]. Alternatively, it has been argued that disability further adds to the power disparities and lack of access resources associated with traditional gender roles [58]. While there is a dearth of methodologically robust studies, it is clear that both gender and type of disability affect the prevalence of violence [11, 58, 59]. It is critical that strategies to prevent violence are developed taking into account these intersectionalities [11, 58, 59].

Overall, the findings of this study highlight the intersectionality between Indigeneity and ableism, which in turn has important implications for the design of services for Aboriginal and Torres Strait Islander people. It is an important complement to narrative research aimed at providing a voice to Aboriginal and Torres Strait Islander people with disability to share their unique experiences and their resilience [20]. National policy for the provision of disability support does include an Indigenous Engagement Strategy; however, the strategy has not been consistently implemented and needs to be considerably strengthened to address the complex needs for Aboriginal and Torres Strait Islander people with disability. This is particularly true in relation to ensuring that disability supports are responsive to the mental and emotional effects of violence, such as trauma or mental illness. Unlike many other sectors (e.g. health, education) there is currently no overarching framework for self-determination, working with Aboriginal and Torres Strait Islander people or cultural safety in the disability sector. This study provides further support to research calling for Aboriginal and Torres Strait Islander people with disability to lead the development of frameworks and approaches for the disability sector [20].

### Study limitations

Our study contains a number of limitations. First, the NATSISS is a cross-sectional survey and we do not make causal inferences about the relationship between the various measures of disability and exposure to violence. Second, the NATSISS operationalises violence as either physical violence or violent threats, omitting specific questions related to sexual assault and sexual threats. Third, the violence measures were collected by self-report over a 1 year period, raising the issue of recall bias. This may lead to an under-estimate of levels of violence which may also exist due to the sensitive nature of the questions. Fourth, as noted earlier, the sampling frame for the NATSISS consisted primarily of people living in private dwellings. Respondents in institutions, for example, were not enumerated in the survey. Finally, Aboriginal people with very severe disabilities may have been under enumerated.

## Conclusions

This study shows that among Aboriginal and Torres Strait Islander people, 1) presence of a disability was associated with a 1.5 fold increase in the odds of exposure to violence and doubling of odds of reporting violent threats, 2) that people with profound or severe disability experienced heightened exposure, in excess of double odds, 3) as were those with specific types of disabilities such as psychological or head injury, stroke and brain damage conditions. We further found that women, regardless of disability status were more likely to report family violence, whereas men were more likely to report violence from other known individuals (i.e., non-partner or familial). Independent of disability status, we further found that family removal was strongly associated with physical violence and violent threats. These findings underscore the unique position of Aboriginal and Torres Strait Islander people with disability, who are at heightened vulnerability of experiencing violence and threats due to overlapping forms of marginalisation and the invisibility of the Indigenous experience of disability. Accessing care is further complicated by the separate Indigenous, violence, and disability services which are difficult to navigate. These results underscore the need to develop policy approaches to ensure that service provision to Aboriginal and Torres Strait Islander people with disability is culturally safe and to ensure that disability support considers the vulnerability of people with disability to violence.

## Data Availability

Data from the National Aboriginal and Torres Strait Islander Social Survey are available to registered users of the Australian Bureau of Statistics microdata.

## Abbreviations

NATSSIS: National Aboriginal and Torres Strait Islander Social Survey
ABS: Australian Bureau of Statistics
SDM: Short disability module
CURF: Confidentialised unit record file
